# Body mass index trajectories from adolescent to young adult for incident high blood pressure and high plasma glucose

**DOI:** 10.1371/journal.pone.0213828

**Published:** 2019-05-01

**Authors:** Noushin Sadat Ahanchi, Azra Ramezankhani, Richard J. Munthali, Samaneh Asgari, Fereidoun Azizi, Farzad Hadaegh

**Affiliations:** 1 Prevention of Metabolic Disorders Research Center, Research Institute for Endocrine Sciences, Shahid Beheshti University of Medical Sciences, Tehran, Iran; 2 Department of Biostatistics and Epidemiology, Research Institute for Endocrine Sciences, Shahid Beheshti University of Medical Sciences, Tehran, Iran; 3 MRC/WITS Developmental Pathways for Health Research Unit, Department of Paediatrics, School of Clinical Medicine, Faculty of Health Sciences, University of Witwatersrand, Johannesburg, South Africa; 4 Endocrine Research Center, Research Institute for Endocrine Sciences, Shahid Beheshti University of Medical Sciences, Tehran, Iran; International University of Health and Welfare, School of Medicine, JAPAN

## Abstract

**Objectives:**

To explore the association between sex-specific adiposity trajectories among Adolescents to early adulthood with incident high blood pressure (HBP) and high plasma glucose (HPG).

**Methods:**

We studied body mass index (BMI) trajectories among1159 (male = 517) and 664 (male = 263) Iranian adolescents, aged 12–20 years, for incident HPG and HBP, respectively. Latent Class Growth Mixture Modeling (LCGMM) on longitudinal data was used to determine sex-specific and distinct BMI trajectories. Logistic regressions were applied to estimate the relationship between latent class membership with HBP and HPG, considering normal trajectory as the reference.

**Results:**

For both HBP and HPG, LCGMM determined two and three distinct BMI trajectories in males and females, respectively. During a follow-up of 12Years 104 (male = 62) and 111(male = 59) cases of HPG and HBP were found, respectively. Among females, faster BMI increases (i.e. overweight to early obese trajectory) but not overweight (i.e. those with BMI = 27.3 kg/m^2^ at baseline) trajectories increased the risk of HPG by adjusted odds ratios (ORs), 2.74 (1.10–5.80) and 0.79 (0.22–2.82), respectively; regarding HBP, the corresponding value for overweight to late obese trajectory was 3.72 (1.37–11.02). Among males, for HBP, the overweight trajectory increased the risk [2.09 (1.04–4.03)]; however, for incident HPG, none of the trajectories showed significant risk.

**Conclusions:**

Among females, trend of increasing BMI parallel with age can be a better predictor for risk of developing HPG and HBP than those with higher BMI at baseline.

## Introduction

Overweight and obesity in adolescents have increased substantially in recent decades and are reported to affect around 30% of adolescents in some developed countries[1]. The rising prevalence of obesity has become a public health crisis among Iranian populations. In a cross-sectional nation-wide study performed in 30 provinces in Iran (2011–2012), among 14880 school students, aged 6–18 years, the prevalence of general obesity was 13.58% and 10.15% in males and females, respectively [2]. Moreover, among Tehranian adolescents, a rising prevalence of both general and central adiposity was reported during 9 years of follow-up; the overall adjusted prevalence of “at risk for overweight” and “overweight” changed from 14.8% and 4.7% in(1999–2001)to 23.0% and 9.2% in (2006–2008)[3]. According to a national study, the prevalence of high plasma glucose(HPG)[type2 diabetes (T2D) or pre diabetes]and high blood pressure(HBP)[hypertension or pre hypertension), among Iranian adults were reported to be 24.5 [4]and 71%, respectively[5]. In fact, among Iranian adults, the population attribute able fraction (PAF) of T2D and hypertension were 24 and 17%, respectively, for all-cause mortality[6].

Understanding the life-course progression of adiposity in children is important since childhood adiposity is associated with adult obesity which is reported to be linked to increased risk of disease or death in later life [7]. The impact of adiposity trajectories from early adolescence with incident hypertension and T2D among young adults has been examined few studies[8–12]. In the present study, we aimed to investigate whether body mass index (BMI) trajectories among Iranian adolescents, contribute independently to the risk of HPG and HBP. We used a Latent Class Growth Mixture Modeling (LCGMM) to identify distinct sex-specific adiposity trajectories. The key advantage of such modeling is that it allows for the estimation of inter-individual variability in intra-individual patterns of change over time. The aims of this study were to: 1) determine the distinct sex-specific patterns of adiposity trajectories in adolescents aged≥12&<20 years, whose BMI changes had been followed for 12 years, and 2) to explore associations of these distinct adiposity trajectories with HPG and HBP.

## Materials and methods

### Study design and sample

The Tehran Lipid and Glucose Study (TLGS) is a large-scale population-based prospective cohort study with long-term follow-up, performed on a representative sample of residents of Tehran. A total of 15005 individuals aged ≥3 years participated in the first examination (1999–2002) of the study, which to date, has been conducted in 5 phases at 3-year intervals from 1999 to 2015. A detailed description of TLGS has been reported elsewhere[13]. For the current study, all subject aged ≥12 and <20 years (n = 2542) were included. First, exclusions included those with missing data on FPG, SBP/DBP and BMI in the baseline (n = 148), those without any follow-ups (n = 556) and missing data on SBP/DBP and FPG in phase5 (n = 624). Missing values on FPG and SBP/DBP at phases 2(2002–2005), 3(2005–2008), 4(2009–2011) were replaced by the Last Observation Carried Forward (LOCF) technique. Secondly, for the analysis of incident HPG, those with prevalent HPG in phases, 2, 3 and4 (n = 203) and regarding incident HBP, those with prevalent HBP at phases 2, 3 and 4 (n = 718) were excluded, resulting in a total of 1159 (males = 517) and 644 (males = 228), participants, respectively, who were followed until phase 5(2012–2015). Informed written consent was obtained from all participants and the ethics committee of the Research Institute for Endocrine Sciences of the Shahid Beheshti University of Medical Sciences approved this study.

### Clinical and laboratory measurements

Using a standard questionnaire a trained interviewer collected information including demographic data, drug history, past medical history of diabetes mellitus. Details of anthropometric measurements including weight and height are reported elsewhere[13]. BMI was calculated as weight in kilograms divided by height in square meters (kg/m^2^). After a 15-minute rest in the sitting position, two measurements of SBP and DBP were measured by trained personnel, on the right arm, using a standardized mercury sphygmomanometer (calibrated by the Iranian Institute of Standards and Industrial Researches); the mean of the two measurements was considered as the participant’s blood pressure (BP). A blood sample was taken between 7:00 and 9:00 AM for all study participants, after 12 to 14 hours of overnight fasting. FPG were measured using an enzymatic colorimetric method with glucose oxidase; inter- and intra-assay coefficients of variations (CV) at baseline and follow-up phases were both less than 2.3%.

### Definition of terms

For adolescents aged 12 to 18 years, BP was defined as either normotensive (BP <90^th^ percentile) or HBP (BP ≥90^th^percentile) for sex, age and height. For participants ≥18 years, the cut-offs recommended by the seventh report of Joint National Committee on Prevention, Detection, Evaluation, and Treatment of HBP (JNC-VII) was applied[14]. Accordingly, pre hypertension was defined as SBP readings from 120 to 139 mmHg or a DBP from 80to89 mmHg while hypertension was defined as SBP ≥140 mmHg or DBP ≥90 mmHg or using anti-hypertension medications. In the current study, we defined HBP as those who have pre-hypertension or hypertension. A HPG concentration was defined as FPG ≥ 100 mg/dl or treated diabetes at baseline.

### Statistical analysis

To identify distinct sex-specific adiposity trajectories, we used LCGMM[15]. Using LCGMM adds more heterogeneity in the model, and thus it was the more flexible method, and was preferred for the current analysis[16–18]. LCGMM is an extension of conventional analyses that we assume all individuals in the study sample come from a homogeneous population; i.e. one (average) trajectory will adequately describe the developmental pattern of the whole sample. This assumption is relaxed in LCGMM, i.e. persons in the sample need not come from one homogeneous underlying population, but can be from multiple, underlying (or latent) sub-populations[19]. Each potential class has its own growth parameters (i.e., intercept and linear slope), where the intercept represents the average BMI-value at baseline and the slope is indicative of the rate at which the BMI values change over time[20].Various LCGMM models were run before we selected a final model. First, various linear LCGMM with fixed intercept and slope variance within classes were investigated. Next, quadratic slopes were added to the model allowing for curved developmental patterns. To determine the optimal number of classes, a common forward procedure was performed, starting with a one class solution(i.e., assuming all individuals follow a similar BMI trajectory over time), and then adding more classes one at a time to investigate whether or not the model fit improves due to the additional class. The number of groups were determined by considering Bayesian information criteria (BIC), entropy, Akaike information criteria (AIC), Adjusted Lo-Mendell-Rubin likelihood ratio test(A LMR test), class size and interpretability[21]. BIC has been shown to be a robust indicator of the preferred k class solution. Lower BIC values indicate a better model fit[22]. The AIC evaluates a model or partition scheme by combining the maximum log-likelihood value with a penalty that depends on the number of parameters being estimated. The models that generate the lowest values are optimal[22]. Entropy is an index used for assessing the precision of assigning latent class membership. Higher entropy values indicate greater precision of classification [16]. An LMR test assesses the differences in the log-likelihood between LCGA models with k and k+1 clusters, where p-values<0.05 indicate the benefit of adding an additional cluster [23]. We also took the posterior probabilities to assign subjects to the trajectories. Each individual in the sample was reassigned to their most-likely class based on these probabilities, which are the highest probability, hence the classes are clearly distinguished from each other. Based on posterior probability, persons were assigned to the trajectory that best matched their work engagement; a probability >0.8is recommended and a probability closer to 1 indicates better classification[21]. Clinical interpretation and class sample size were also considered in the decision making process. We rejected models with clinically uninterruptable classes and classes with <1% of the total sample[16]Missing data were adequately handled by the Expectation-Maximization algorithm which is an iterative method used to find maximum likelihood estimation with robust standard errors by missing at random (MAR) assumption[16, 24]. Finally, to estimate the relationship between latent class membership with HPG and HBP, both unadjusted and multivariate logistic regressions were used, considering normal weight trajectory as reference. The related adjusted covariates were selected bases on previous literature. For incident HPG, the model was adjusted for age, family history of diabetes, SBP and DBP; only age was the corresponding cofounder for incident HBP. Baseline characteristics of different trajectories were reported by their mean (standard deviation; SD) or frequency (percentage; %) for continuous and categorical variables, respectively. Statistical significance of the differences among different categories was tested using ANOVA and Chi square test as appropriate. Furthermore, the outliers of BMI were checked by values 3 standard deviations (SD) ±beyond the mean S1 Fig. Statistical analyses were conducted using the Mplus 7.4 and Stata 14.0 [25]. Analysis was sex stratified and p-value of <0.05 was considered significant.

Now the model can be presented as:

$$f(y)=\sum_{k=1}^{K}\mathrm{Pr}\left(C=k\right)\mathrm{Pr}\left(Y=y|C=k\right)=\sum_{k=1}^{K}p_{k}f(y,\lambda_{k})$$ (1)

The main goal in the model in Eq (1) is to estimate p_k_, which is the probability of an individual belonging to class k, BMI development pattern, as a function of parameter λ_k_, where λ_k_ dependent on time.F(y) can be a linear, quadratic or cubic function. We implement this model in Mplus software and see the model fitting parameters to choose the best fitting growth function, even before determining the number of classes; hence, only quadratic models involving quadratic time function were further analyzed.

## Results

### BMI trajectories for incident HPG

The study population included 517 and 642 males and females, mean (SD) age 15.46(2.18) and15.64 (2.26), respectively. After applying criteria as recommended by other authors[22, 26], only quadratic models involving quadratic time function were further analyzed.Table 1 shows the model fit statistics for the growth mixture model with 1 to 6classes. In both genders, the 4- and 5-class models resulted in some classes that included less than 1% of the sample and were hence excluded from further consideration; therefore, the 3-class model was chosen as the optimal class size because it had the lowest BIC, sample size adjusted BIC and AIC among the remaining models.

**Table 1:** Model fit statistics for quadratic LCGMM comparing classes 1 to 6 latent for incident HPG.

Number of classes	Number of parameters	AIC	BIC	ABIC	Entropy	A LMR test P value	
Females (n = 642)							
**Two**	18	12940.62	13020.98	12963.83	0.88	0.03	
Three*	22	12904.01	13002.23	12932.38	0.83	0.05	
**Four**	26	12876.95	12993.03	12925.48	0.83	0.27	
**Five**	30	12859.44	12993.38	12898.13	0.82	0.45	
**Six**	34	12849.36	13001.16	12893.21	0.88	0.48	
Males (n = 517)							
Two*	18	9842.62	9919.08	9861.95	0.86	0.05	
**Three**	22	9789.86	9883.32	9843.49	0.87	0.15	
**Four**	26	9757.75	9868.20	9785.67	0.91	0.17	
**Five**	Not identified					

**HPG**: High plasma glucose; **LCGMM**: latent class growth mixture modeling; **AIC**: Akaike information.criterion; **BIC**: Bayesian Information Criterion; **ABIC**: Adjusted Bayesian Information Criterion; **A LMR test.** Adjusted Lo-Mendell-Rubin likelihood ratio test. Lower BIC, AIC values indicate better fit. Higher entropy values indicate greater precision of class membership assignments.

*The optimal class number according to the model fit criteria.

**Fig 1:**
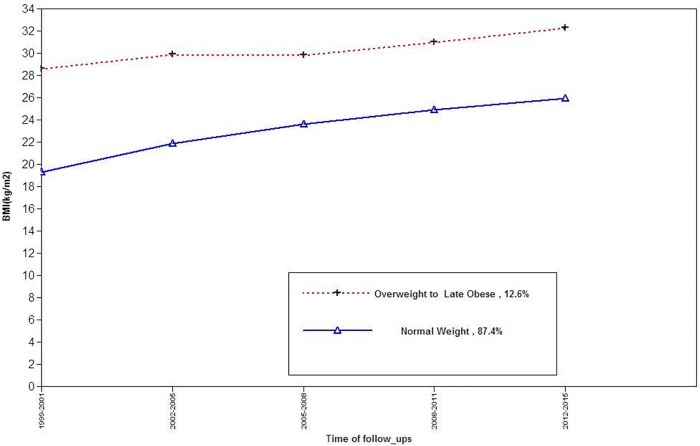
BMI trajectories among females, for the outcome of incident high plasma glucose.

**Fig 2:**
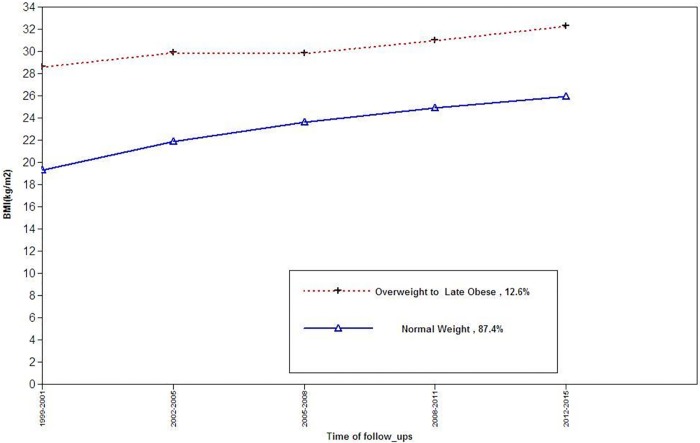
BMI trajectories among males, for the outcome of incident high plasma glucose.

For females, three BMI trajectory classes can be described as trajectory 1(normal, n = 539, 82.9%) and Trajectory2 (overweight to early obese, n = 59, 9.2%) and trajectory 3(overweight, n = 51 7.9%) (Fig 1).For males, the two BMI trajectory classes can be described as: Trajectory 1(normal weight, n = 454, 87.8%) and trajectory 2(Overweight to obese, n = 63, 12.2%) (Fig 2).Characteristics of study population by BMI trajectory classes are shown in Tables 2 and 3 In each gender, SBP and DBP, age, and the level of BMI as well as incident HPG differed significantly different among BMI trajectories. At the end of follow-up, we found 104 cases (males = 62) with incident HPG; of these, 1 male and 10 females were taking glucose-lowering drugs. As shown in Table 4, in multivariate analysis, among females, compared to the reference, overweight to early obese trajectory was significantly associated with HPG [odds ratio (OR) 2.74 (95% confidence interval (CI): (1.10–5.80)], whereas, the overweight trajectory did not show any such risk [1.01 (0.29–3.42)].Among males, no association was found for different trajectories.

**Table 2:** Baseline characteristics of study population by BMI trajectories for incident HPG.

Females	Trajectory 1 (n = 532)	Trajectory 2 (n = 59)	Trajectory3 (n = 51)	P value
Age (years)	15.2 (2.26)	16.14 (2.27)	16.3 (2.03)	0.02
Fasting plasma glucose (mg/dl)	85.9 (7.03)	84.6(7.9)	85.80 (7.28)	0.58
BMI (kg/m^2^)	19.7 (2.62)	25.7 (2.27)	27.6(1.91)	0.00
Family history diabetes, n (%)	7 (9.9)	6 (11.1)	48 (14.3)	0.40
SBP (mmHg)	103.5(10.59)	110.0(11.40)	111.9(11.3)	0.00
DBP (mmHg)	70.9(8.99)	75.2(9.53)	75.7(7.06)	0.00
HPG, n (%)	31 (5.8)	8 (13.7)	3 (5.9)	0.008
Mean Posterior Probabilities	0.94	0.88	0.95	0.00

Data are mean (standard deviation) for continues, and frequency (%) for categorical variables

**BMI:** Body mass index; **DBP**: Diastolic blood pressure; **SBP:** Systolic blood pressure; **HPG:** High plasma glucose

Family history diabetes defined as having at least one parent or sibling with diabetes.

**Table 3:** Baseline characteristics of study population by BMI trajectories for incident HPG.

Males	Trajectory 1 (n = 454)	Trajectory 2 (n = 63)	P value
Age (years)	15.3 (2.19)	16.5(1.73)	0.00
Fasting plasma glucose(mg/dl)	87.3(6.7)	88.8(5.63)	0.09
BMI (kg/m^2^)	19.28(2.41)	29.00 (3.25)	0.00
Family history diabetes n (%)	54 (11.3)	13 (20.5)	0.70
SBP (mmHg)	106.1(11.26)	119.2(10.49)	0.00
DBP (mmHg)	70.4 (9.36)	76.9 (8.03)	0.00
HPG, n (%)	55 (12.1)	7(11.1)	0.96
Mean Posterior Probabilities	0.96	0.90	0.00

Data are mean (standard deviation) for continues, and frequency (%) for categorical variables

**BMI:** Body mass index; **DBP**: Diastolic blood pressure; **SBP:** Systolic blood pressure; **HPG:** High plasma glucose

Family history diabetes defined as having at least one parent or sibling with diabetes.

**Table 4:** Associations between BMI trajectories and HPG.

Females	Crude OR (95% CI) *	Adjusted OR (95% CI) †
Normal weight trajectory	Reference	Reference
Overweight to early obese trajectory	2.53(1.10–5.80)	2.74 (1.10–5.80)
Overweight trajectory	1.01(0.29–3.42)	0.79 (0.22–2.82)
Age (years)	…….	1.00 (0.87–1.16)
Family history of diabetes	…….	1.74 (0.72–4.23)
SBP (mmHg)	………	1.03 (1.00–1.07)
DBP (mmHg)	……..	0.99 (0.96–1.04)
**Males**		
**Normal weight trajectory**	Reference	Reference
Overweight to late obese trajectory	1.10 (0.47–2.54)	1.07 (0.58–2.63)
Age (years)	………	1.11 (0.97–1.27)
Family history of diabetes	……..	0.86 (0.38–1.91)
SBP (mmHg)	…….	1.00 (0.97–1.03)
DBP (mmHg)	……..	0.97 (0.93–1.005)

Family history of diabetes defined as having at least one parent or sibling with diabetes; **CI**: confidence interval; OR: odds ratio; **DBP**: Diastolic blood pressure; **SBP:** Systolic blood pressure; **HPG:** High plasma glucose

* Unadjusted models

**†** Models were adjusted for age, family history of diabetes, DBP and SBP

The power of overweight to late obesity trajectory and overweight to obese trajectory considering Normal weight as reference group in the adjusted model are …28%, 30%,. respectively.

### BMI trajectories for incident HBP

The study population included 228males and 416females, mean (SD) age of 14.94(2.13) and 15.48(2.30) years, respectively. Results of LCGMM analysis revealed that quadratic terms function was best fitted compared the linear model. Table 5 shows the quadratic model fit statistics for 1 to 6classes. As this table shows, the 4- and 5-class models included less than 1% of the females and hence were excluded from further consideration. Hence the 3-class model was chosen as the optimal class due to the lowest BIC, sample size adjusted BIC and AIC between the remaining models. For males, a 2-class model had the best fit.

**Table 5:** Model fit statistics for quadratic LCGMM comparing 1 to 6 latent classes for incident HPB.

Number of classes	Number of parameters	AIC	BIC	ABIC	Entropy	A LMR test P value
**Females** **(n = 445)**						
Two	18	8090.63	8167.18	8110.06	0.94	0.2
Three	22	8066.72	8155.39	8085.58	0.91	0.5
Four	26	8042.90	8147.70	8065.20	0.85	0. 7
Five	30	8033.99	8154.91	8059.71	0.85	0.3
Six	34	8027.14	8164.18	8056.29	0.85	0.29
**Males** **(n = 263)**						
Two* Three	18	4009.01	4070.74	4013.69	0.77	0.55
Four						
five and six	Not identified	-	-	-	-	-

**HPB:** High plasma pressure; **LCGMM**: Latent class growth mixture modeling; **AIC**: Akaike information criterion; **BIC**: Bayesian Information Criterion; **ABIC**: Adjusted Bayesian Information Criterion; **A LMR test:** Adjusted Lo- Mendell-Rubin likelihood ratio test Lower BIC, AIC values indicate better fit. Higher entropy values indicate greater precision of class membership assignments.

**Fig 3:**
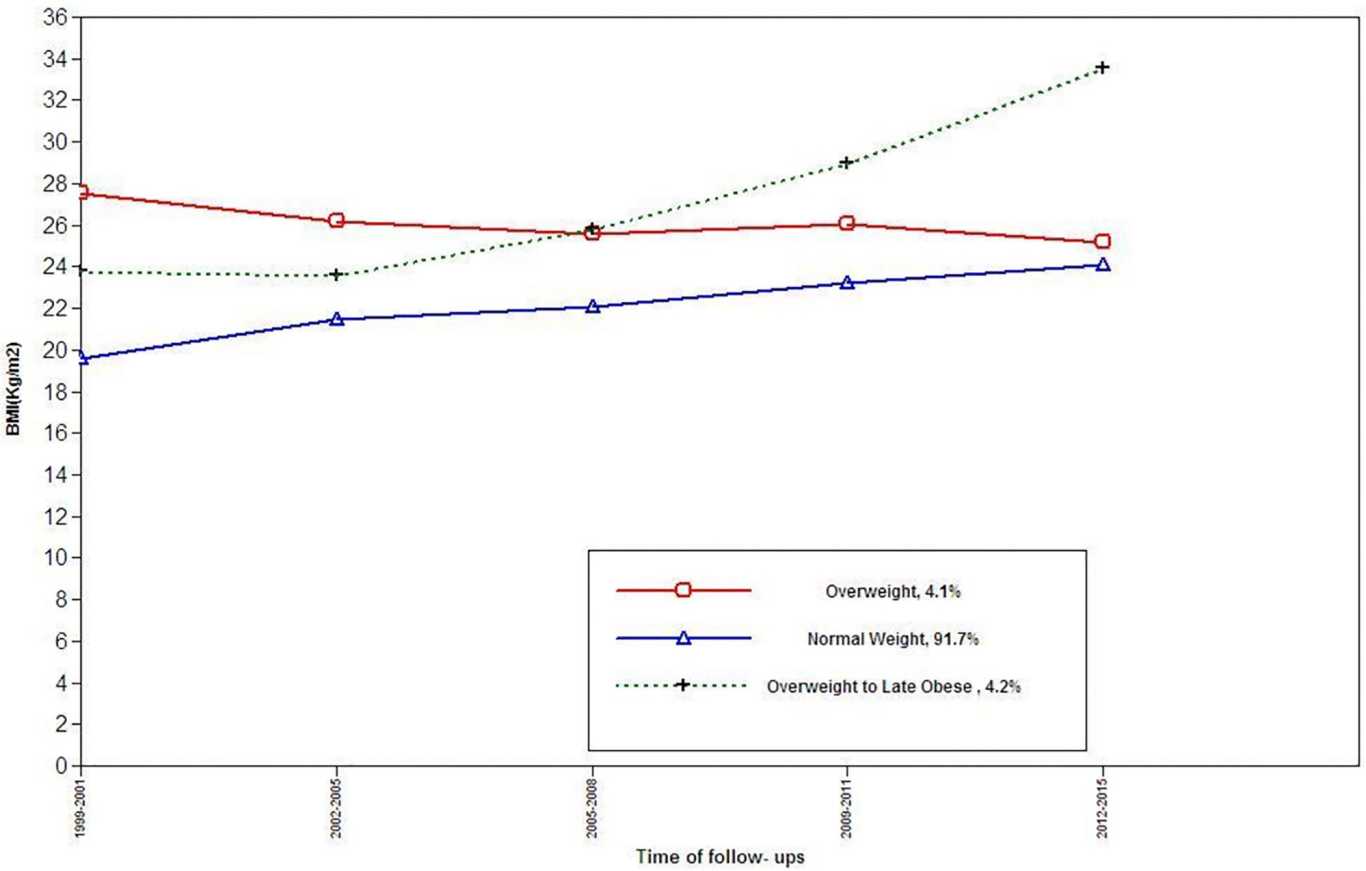
BMI trajectories among females, for the outcome of incident high blood pressure.

**Fig 4:**
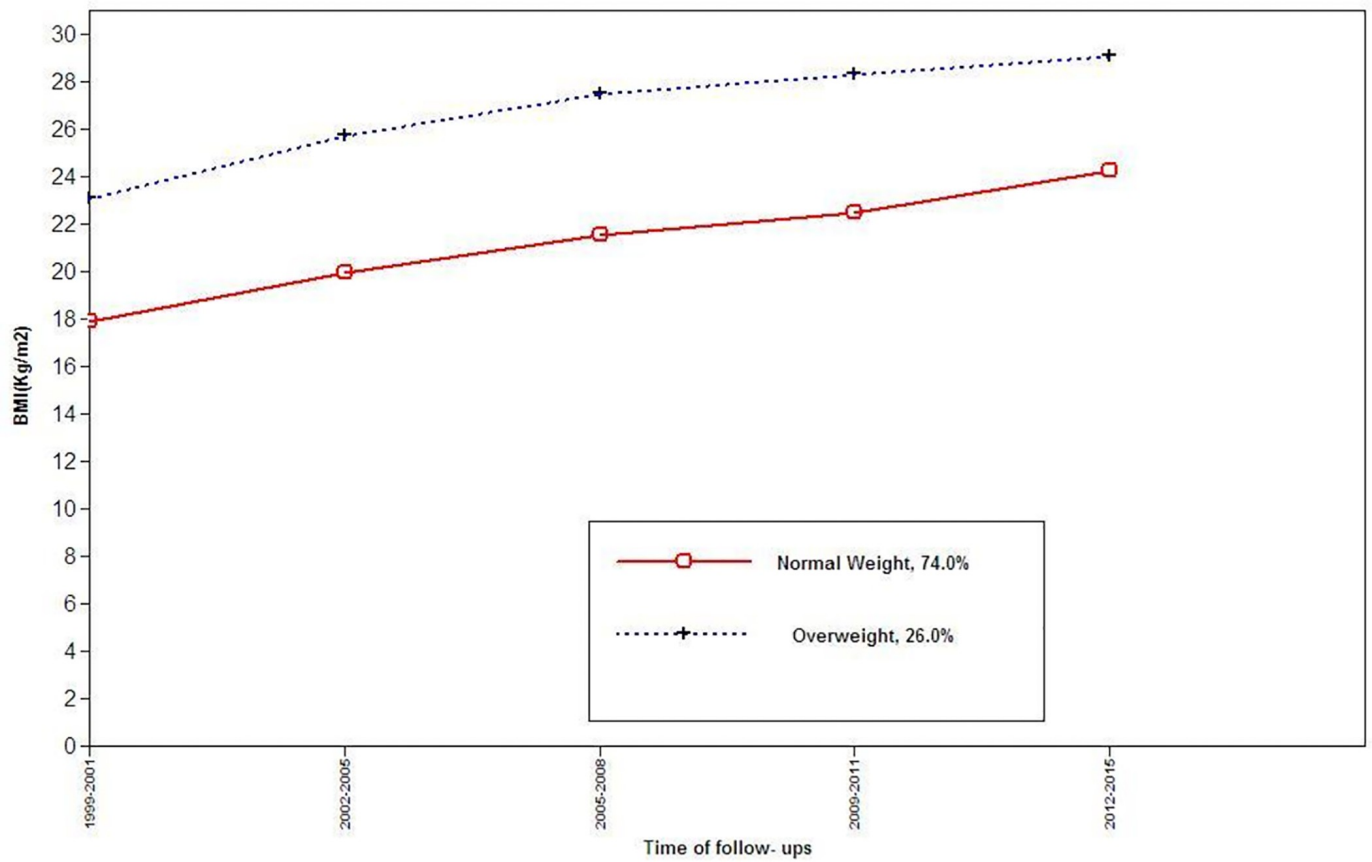
BMI trajectories among males, for the outcome of incident high blood pressure.

Among females, three BMI trajectory classes were identified: trajectory 1(normal weight, n = 386, 92.8%), trajectory 2 (Overweight to late obesity, n = 15, 3.6%), and trajectory 3(Overweight, n = 15, 3.6%) (Fig 3); while, in males the two BMI trajectory classes were detected: trajectory 1(normal weight, n = 171, 75%), and trajectory 2(Overweight, n = 57, 25%) (Fig 4). Baseline characteristics of participants by BMI trajectory classes are shown in Table 6; in both genders, BMI and the incident HBP were significantly different between trajectories, however, only among males, the statistical differences in SBP and DBP was found. At the end of follow-up, 111 participants (59 males) with incident HBP were identified. Results of multivariate analysis among females are shown in Table 7. Compared to reference, the overweight to obesity trajectory was significantly associated with HBP [3.72 1.37–11.02)]; among males compared to reference the overweight trajectory was associated with incident HBP [2.09 (1.04–4.03)].

**Table 6:** Baseline characteristics of study population by BMI trajectories for incident HBP.

**Females**	**Trajectory1** **(n = 386)**	**Trajectory 2** **(n = 15)**	**Trajectory 3 (n = 15)**	**P value**
Age (years)	15.46(2.32)	15.33 (2.19)	16.2(2.18)	0.31
BMI(kg/m^2^)	19.60(2.86)	24.1(3.6)	28.2(2.12)	0.00
SBP (mmHg)	100.7(9.47)	104.3(8.64)	104.3 (9.64)	0.27
DBP(mmHg)	67.7(7.15)	68.8 (8.04)	70.75(6.41)	0.21
HBP, n (%)	47 (12.2)	5 (33.3)	0.00 (0.00)	0.007
Mean Posterior Probabilities	0.97	0.88	0.89	
**Males**	**Trajectory1** **(n = 171)**	**Trajectory2** **(n = 61)**		**P value**
Age (years)	14.7 (2.1)	15.4 (2.11)		0.08
BMI(kg/m^2^)	17.8 (1.83)	23.5 (2.4)		0.00
SBP(mmHg)	100.2 (10.11)	104.7(9.2)		0.002
DBP(mmHg)	65.6(7.34)	68.1(6.55)		0.01
HBP, n (%)	38 (22.8)	21(36.8)		0.04
Mean Posterior Probabilities	0.96	0.88		

Data are mean (standard deviation) for continues, and frequency (%) for categorical variables. **BMI**: Body mass index, **DBP**: Diastolic blood pressure, **SBP**: Systolic blood pressure; **HBP**: high blood pressure

**Table 7:** Associations between BMI trajectories and HBP.

	OR (95% CI) *	Adjusted OR (95% CI) †
**Females**		
Normal weight	Reference	Reference
Overweight to late obesity trajectory	3.60 (1.18–11.00)	3.72 (1.37–11.02)
Overweight trajectory	1^#^	1
Age (years)	-	1.12 (0.98–1.27)
**Males**		
Normal weight	Reference	Reference
Overweight trajectory	2.04 (1.06–3.90)	2.09 (1.04–4.03)
Age (years)	-	0.96 (0.83–1.1)

**CI**: Confidence interval, **OR**: Odds ratio; **BMI**: Body mass index, **HBP**: High blood pressure; **HPG:** High plasma pressure.

#In this Trajectory no any case of HBP was found. The power of overweight to late obesity trajectory considering, normal weight as the reference group in adjusted model is 30%…

* Unadjusted models

**†** Models were adjusted for age

### Cluster membership uncertainty

It is important to note that in LCGMM analysis, class membership is not determined with certainty for each individual; rather each individual has a probability of belonging to one of the latent classes. Thus class membership uncertainty should be taken into account in studying the association of individual characteristics with the latent classes., in the current analysis, the mean posterior probability for each identified trajectories was reported; all of these were above 0.85 (Min posterior = 0.88). Also the percentage of individuals who had (probability>0.80 and probability<0.80) for each identified trajectories are also reported (Tables 2 and 6). Thus, with so few cases with moderate and none with substantial uncertainty with this particular solution, we believe there is limited bias derived from selecting the most likely cluster membership for each individual.

## Discussion

In the current study conducted among Iranian adolescents followed for over a decade, we examined the developmental patterns of BMI in relation to incident HBP and HPG during early young adulthood. Our results confirm that there is heterogeneity in BMI trajectories in the study sample and that these trajectories vary between males and females. Among females, the three-class BMI trajectory model may best represent heterogeneity in BMI developmental patterns for incident HPG and HBP. Compared to the normal weight trajectory, for HPG, the overweight to early obese trajectory increased the risk approximately 3-fold; for HBP, the corresponding significant risk for overweight to late obese trajectory was about 4-fold.Among males, the two class BMI trajectory models may best represent heterogeneity in BMI developmental patterns for incident HPG and HBP. Compared to normal weight trajectory, for HBP the overweight trajectory increased the risk by about 2- fold; however, for incident HPG, none of the trajectories showed significant risk.

In BMI trajectories, the heterogeneity we observed between males and females for incident HPG and HBP has also been reported by other researchers [9, 27, 28].In line with our findings regarding HBP, Munthaliet al. in a South African urban township among a population aged, 5–18 years found heterogeneity in BMI trajectories between genders [9]. Similarly a study by Hejazi et al. of Canadian children aged 2–8 years [28] and a study by Haga et al. reported that BMI trajectories varied between males and females in Japanese children aged between birth and 12 years[27]. In males, for incident HPG, those with BMI >35 kg/m^2^ at baseline was associated with faster declines and earlier rebounds in BMI, suggesting that these males undergo the most intense changes in physical growth during this developmental period, although this trajectory was not a predictor for HPG. This is consistent with CDC and WHO growth charts, which depict faster declines and more rapid rebounds for males in higher BMI percentiles. For incident HBP, we found that being in an overweight trajectory was associated with increased risk of incident HBP in males, which is in agreement with the study of Munthaliet al (8). In females, faster BMI increases were strongly associated with incidence of HPG risk for young adults. Also, in adolescents with higher slope, (the overweight to early obese and overweight to late obese trajectories) was significantly associated with the risk of developing HPG and HBP compared to adolescents with higher BMI at baseline (overweight trajectory), despite the fact that both groups had early onset of overweight. In other words, in our study the trend of increasing BMI in parallel with age in females can be a better predictor for risk of developing HPG and HBP compared to adolescents with higher intercept BMI. Increasing levels of BMI might be a harbinger of insulin resistance status which is a main contributor to incident hypertension and diabetes [29, 30]. This could be important in reducing hypertension and diabetes risk early in adulthood. We found that being in an overweight to late obese was associated with increased risk of HBP in females, which is in agreement with other studies[9, 31].

The mechanisms of the sex differences observed in the impact BMI trajectories for incident HBP and HPG in our study and other studies in this field are not clear [9, 27, 32]; and may be attributed to cardiovascular fetal programming as reported earlier[33, 34]. In fact, the exposure to intrauterine hyperglycemia and other factors linked with maternal obesity may be of greater relevance than the genetic susceptibility[35]. As for diabetic mothers, there are some important sex-related differences in the sensitivity of the children to maternal metabolic overload with higher susceptibility of the girl fetus[36]. Of special interest in this context there are gender differences in the offspring regarding the severity and age-dependent onset of the disease as well as the role of sex hormones in mediating the observed sexual dimorphism in response to changes in intrauterine environment[33].

Remarkably, for incident HBP, a significantly elevated risk was observed only in the overweight to late obese trajectory in females (i.e. OR≈ 4), suggesting that after a relatively short time of about 3 years after initiation of obesity, significant risk for HBP was found as compared with the impact of overweight to early obese trajectory (i.e. OR≈ 3) for incident HPG which needs to have an obesity status for about 9 years for development of this outcome. Among adolescent females, we hence speculate that a longer duration of obesity is needed for development of HPG rather than HPB.

The current study has several strengths; first, the longitudinal study with long-term follow-up, performed on a representative sample of residents of Tehran. Second, using LCGMM indentifying trajectories is an ideal analytical method since it involves a number of criteria in selecting the best fitting model and predicts individual class membership from the available data and its ability to deal with missing at random assumption. Third, the study examined the impact of trajectories for both HBP and HPG in both genders from early adolescence to young adulthood. Few studies have examined whether there are different trajectories in boys and girls, investigating trajectories in just women only[32, 37] or in men and women combined[37–42]. Araújo and colleagues reported that Increases in adiposity, particularly from late adolescence-to-young adulthood, were associated with cardio metabolic factors in early adulthood[43]. These results are consistent with our result. Nonnemaker et al. reported that those who enter adolescence as overweight are a heterogeneous group in terms of subsequent growth patterns[42], These results are consistent with our result. This is consistent with the literature on tracking[7]. Despite these strengths, the present study is not without limitations. Due to the limited sample size, we might not be able to capture all trajectories, which also influenced the observation that the high-risk trajectory in males comprised of very few individuals that might influence association analysis.

### Conclusions

In this study, we identified distinct sex-specific BMI trajectories. In females, the overweight to early obese and overweight to late obese trajectories were associated with incident HPG and HPB. Among males, overweight trajectory increased the risk of HBP although; no risk was shown for different trajectories in the prediction of HPG. In females, faster BMI increases were strongly associated with incidence HPG and HPB risk for young adults. Also individuals with higher slope (overweight to late obese and overweight to early obese) were significantly associated with the events with higher BMI at baseline (overweight trajectory) despite the fact that both groups had early onset overweight. These results confirm the considering patterns of BMI development, especially at early stages of development, so that prevention strategies may be implemented to target those individuals with high-risk developmental patterns.

## Supporting information

S1 Fig The scatter plot of body mass index over systolic and diastolic blood pressure in male (a) and female (b) for incidence of HBP. (DOCX)

## Abbreviations

BMI: Body Mass Index
LCGMM: Latent Class Growth Mixture Modeling
HBP: High Blood Pressure
HPG: High Plasma Glucose
T2D: Type 2 Diabetes
PAF: Population Attributable Fraction
TLGS: Tehran Lipid and Glucose Study
FPG: Fasting Plasma Glucose
LOCF: Last Observation Carried Forward
SBP: Systolic Blood Pressure
DBP: Diastolic Blood Pressure
BP: Blood Pressure
