# Supplementary material for: Body mass index trajectories from adolescent to young adult for incident high blood pressure and high plasma glucose
Source: PLoS One. 2019 May 1;14(5):e0213828. doi: 10.1371/journal.pone.0213828 (PMC6493705; doi:10.1371/journal.pone.0213828)
Supplement: S1 Fig — (DOCX) [file pone.0213828.s001.docx]

| S1 Fig: The scatter plot of body mass index over systolic and diastolic blood pressure in male (a) and female (b) for incidence of HBP. | | |
| --- | --- | --- |
| **Men** | | |
| a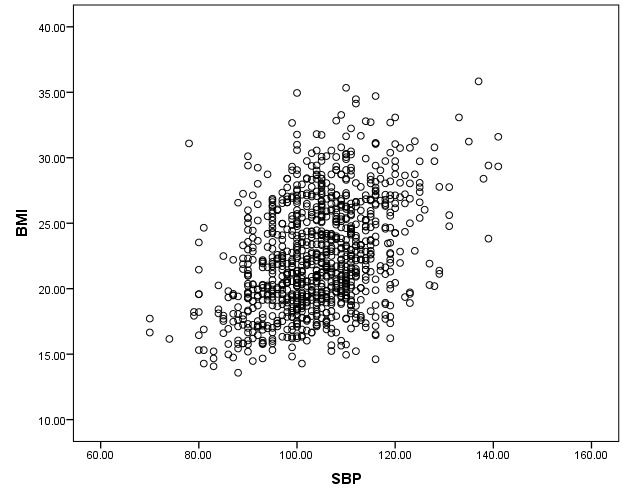 | | 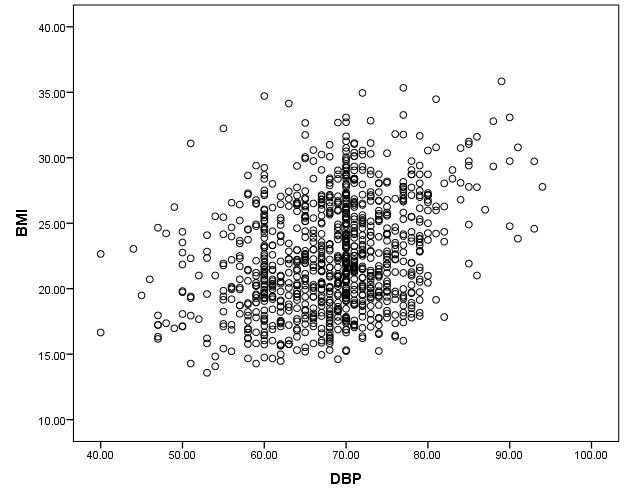 |
| **Women** | | |
| 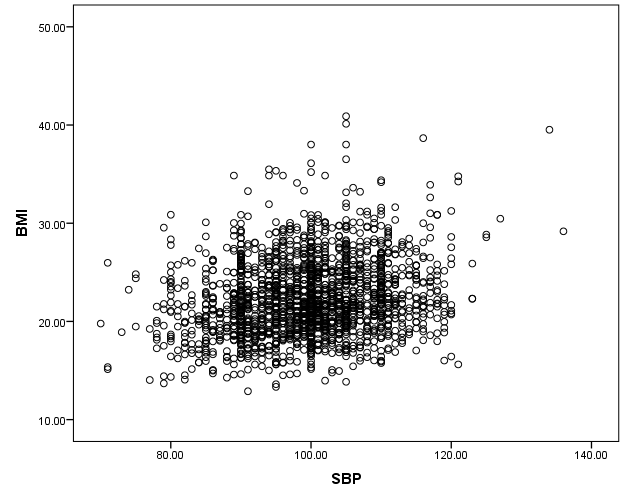 | 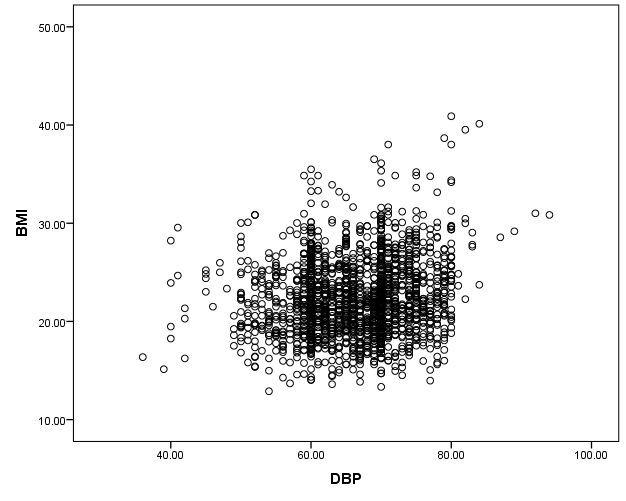 | |
| Time varying exposures were considered; each BMI was assigned to the related SBP/DBP.  BMI: Body mass index; SBP/DBP: systolic/diastolic blood pressure;HBP: high blood pressure | | |
